# A review of machine learning methods for cancer characterization from microbiome data

**DOI:** 10.1038/s41698-024-00617-7

**Published:** 2024-05-30

**Authors:** Marco Teixeira, Francisco Silva, Rui M. Ferreira, Tania Pereira, Ceu Figueiredo, Hélder P. Oliveira

**Affiliations:** 1 Institute for Systems and Computer Engineering, Technology and Science, Porto, Portugal; 2 Faculty of Engineering, University of Porto, Porto, Portugal; 3 Faculty of Science, University of Porto, Porto, Portugal; 4 Ipatimup - Institute of Molecular Pathology and Immunology of the University of Porto, Porto, Portugal; 5 Instituto de Investigação e Inovação em Saúde, University of Porto, Porto, Portugal; 6 Faculty of Sciences and Technology, University of Coimbra, Coimbra, Portugal; 7 Faculty of Medicine, University of Porto, Porto, Portugal

**Keywords:** Computational biology and bioinformatics, Microbiology, Cancer, Mathematics and computing

## Abstract

Recent studies have shown that the microbiome can impact cancer development, progression, and response to therapies suggesting microbiome-based approaches for cancer characterization. As cancer-related signatures are complex and implicate many taxa, their discovery often requires Machine Learning approaches. This review discusses Machine Learning methods for cancer characterization from microbiome data. It focuses on the implications of choices undertaken during sample collection, feature selection and pre-processing. It also discusses ML model selection, guiding how to choose an ML model, and model validation. Finally, it enumerates current limitations and how these may be surpassed. Proposed methods, often based on Random Forests, show promising results, however insufficient for widespread clinical usage. Studies often report conflicting results mainly due to ML models with poor generalizability. We expect that evaluating models with expanded, hold-out datasets, removing technical artifacts, exploring representations of the microbiome other than taxonomical profiles, leveraging advances in deep learning, and developing ML models better adapted to the characteristics of microbiome data will improve the performance and generalizability of models and enable their usage in the clinic.

## Introduction

Cancer is the second most common cause of death worldwide, with 10M estimated deaths in 2020^1,2^. It is a heterogeneous group of diseases that result from complex gene-environment interactions. There is increasing evidence that in addition to the series of hallmarks featured by cancer, the microbiome can have an important impact on cancer phenotypes^3^. The human microbiome is the community of all microorganisms that colonize the human body, including bacteria, viruses, and fungi^4^. The advent of next-generation sequencing (NGS)^5^ technologies made it possible to study the composition of the human microbiome. The widespread use of *omics* approaches^6,7^, together with efforts such as the Human Microbiome Project^8^, have largely improved our understanding of the impact of the microbiome in health and disease. Knowledge about human-microbial interactions is greater for the microbiome of the gut, since it is easy to non-invasively characterize the microbial communities in fecal samples^9^. Changes in the gut microbiome have been associated with various diseases, including inflammatory bowel disease (IBD)^10^, obesity^10^, colorectal cancer^11^, and even anxiety and depression^12^.

Considering the relationship between the microbiome and cancer, a growing amount of clinical and preclinical studies have revealed that the microbiome can impact cancer development, cancer progression, and response to cancer therapies^13^, suggesting microbiome-based diagnostic, prognostic, and therapeutic approaches. It is now clear that the microbial communities found in the tumor tissues and in the oral and gut microbiomes of patients with various types of cancer are distinct from those found in healthy control individuals^14–16^. Studies that indirectly derived microbial profiles from human genome and transcriptome samples of large databases, such as The Cancer Genome Atlas (TCGA)^17^, also identified cancer type-specific microbiota signatures^18,19^. Furthermore, the microbiome found in cancer patients is predictive of their response to chemotherapy and to cancer immune checkpoint therapy^20–22^.

Machine Learning (ML) models are algorithms that make predictions given a dataset of learnable examples. These models can often uncover complex patterns more easily than human experts^23^. Methods based on ML are now ubiquitous in healthcare, with models used in imaging, to manage electronic health records, and for genetic analysis^24^. As microbiome-based signatures are complex and involve multiple species, ML approaches are an optimal tool for the investigation of relationships between the microbiome and host phenotypes^9^. As such, various studies have used ML models for disease prediction based on the gut microbiome^25,26^. Until recently, most of the applications of ML in the field of microbiome and cancer have focused on colorectal cancer and the gut microbiome^27–29^. However, recent studies using ML approaches have suggested biomarkers for cancer detection, disease prognosis, and response to treatment in different cancer types based on the tumor, gut, and oral microbiomes^18,30–35^. Despite their promising results, these approaches require further improvement in the accuracy of models before widespread use and translation of the findings into the clinic.

This review characterizes the state-of-the-art ML approaches for cancer characterization from microbiome data, analyzing all development steps from sample extraction to inference. It tries to bridge the gap between those familiar with ML and researchers experienced in the microbiome field, and help the development of robust approaches by highlighting the strengths and pitfalls of methods. Zhou and Ghallins^36^ previously reviewed ML models for host trait prediction, without focusing on cancer, while Cheung and Yu^37^ focused only on gastrointestinal cancer; however, due to their complexity and recency, cancer-related applications raise challenges that grant a specific discussion. Therefore, this review focuses its analysis on applications linked to the characterization of multiple types of cancer. Furthermore, we aim to analyze ML methods and their performance in greater detail.

**Fig. 1:**
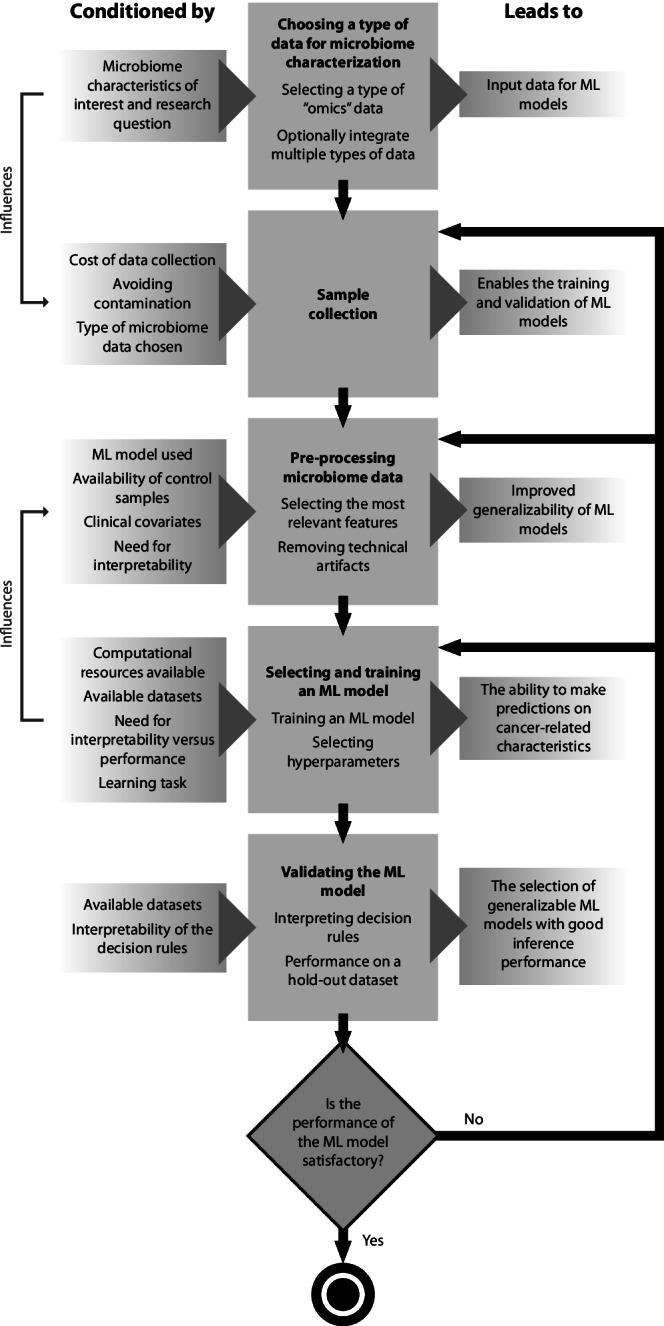
Steps and decisions undertaken when developing an ML model for cancer characterization with microbiome data. The blocks on the left show the factors conditioning each step (represented in the middle blocks). Some of the decisions are also affected by choices made upstream - for instance, the characteristics of the ML model chosen affect how the microbiome data should be pre-processed; these dependencies are represented by the connectors on the left. The blocks on the right represent the contribution of each step to the overall goal of model development and validation. Each step is discussed in detail in this review.

We searched PubMed, Scopus, and IEEE Xplore for relevant publications from 1 January 2015 and up to 1 May 2023. Details on the criteria for inclusion are available in Supplementary Methods 1; Supplementary Fig. 1 contains the number of articles included in each stage of selection. Supplementary Table I holds all full-text articles assessed, characterized by the ML method used. In this manuscript, we start by discussing the sample collection and processing strategies for microbiome characterization, and how to address contamination. Then, we assess the possible input microbiome-derived features for an ML model and feature selection and transformation methods used in this domain. We analyze the most popular ML models and promising deep learning approaches, as well as how to evaluate and validate the ML models developed; we address current limitations, discussing why ML-based findings regarding the association between cancer and the microbiome are often contradictory. Finally, we present future research directions and discuss how to circumvent some of the current pitfalls. Figure 1 contains an overview of all these steps needed to develop and validate an ML model using microbiome data for cancer characterization.

## Sample collection, processing, and decontamination

There are various methods for microbiome characterization, but the most widely used are based on nucleic acids. In these methods, the molecular analysis of the microbiome starts with the acquisition of genetic material from a sample. DNA or RNA isolation can be achieved from several sources, such as fecal samples, mucosal swabs, tissue biopsies, and blood. The selection of sample type is important, as different sources can provide different information about the microbiome and its interactions with the human host. For example, tumor biopsies enable the characterization of the local microbiome, which can provide information on how the microbiome directly influences tumor development or progression. On the other hand, sampling the microbiome at a distant location from the tumor can be indicative of dysbiosis in cancer patients but may not provide information on the interaction between the microbiome and the tumor. Some sample types are easier to acquire than others. Fecal, oral, and blood samples are easy to collect, as they can be sampled with non-invasive or minimally invasive methods^9^, whereas tissues or biopsies require invasive procedures, making sample collection more difficult and demanding.

All types of samples are prone to external contamination with microorganisms, which alter the microbiome composition of the sample leading to biased results. Two main types of contaminations can influence microbiome studies: contamination by external DNA (due to contaminants from the local environment where samples are collected, and during nucleic acid isolation and amplification) and cross-contamination during sample processing. Contaminations can be controlled by acting on two levels: during sample processing and during sequencing data analysis. To account for the potential external contamination, a series of control samples can be prepared by adding all reagents but no genetic material. During sequencing data analysis, the removal of microbial contaminations can be performed by comparing sample microbial content with that observed in the control samples. Extensive decontamination after sequencing has been performed when mining sequencing datasets of the TCGA project for reads of microbial origin. For example, The Cancer Microbiome Atlas (TCMA) provided curated and decontaminated microbiota profiles for oropharyngeal, esophageal, gastrointestinal, and colorectal cancer tissues, after decontamination of species equiprevalent across sample types^38^. In other cases, contaminants were identified by comparing lists of commonly found microorganisms in laboratory reagents and kits with those identified in samples^18^. Other contaminants may be identified by assuming that the abundance of a contaminant is inversely correlated with sample concentration: if the sample concentration is small but the abundance of an organism is unusually high, that organism is likely to be a contaminant^39^.

These in silico approaches do not replace well designed datasets for microbiome analysis, with adequate controls and minimal handling of samples. One study implementing such measures in breast cancer biopsies demonstrated their efficacy, as only low and moderate levels of contamination were found^30^. Stringent decontamination may also improve the separability of breast tumor and normal tissue samples, decreasing the similarity in the feature space between samples from the same patient^30^. This sampling procedure, specific for microbiome analysis of cancerous tissue, is the exception rather than the rule. Still, for most cancer types, bespoke datasets are not available, and their usage may provide more reliable results. In conclusion, when choosing the type of sample to use in cancer-related microbiome analysis, one must consider the research question and the information the sample to be collected may provide. Furthermore, care should be taken to avoid contamination during sample collection and processing. Microbiome data generated from clinical samples should clearly describe all steps from sample processing to analysis and the types of controls included to handle possible contaminants to properly develop and validate models for diagnosis and characterization.

## Types of data for analyzing the microbiome

**Fig. 2:**
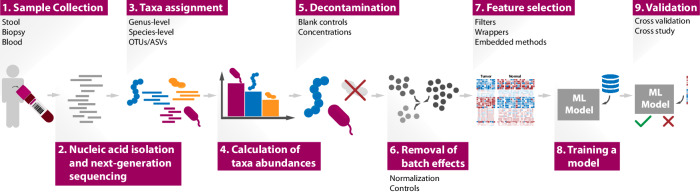
Pipeline for ML-based cancer identification from microbial abundance profiles. The nucleic acid in the collected samples is sequenced and the resulting reads are assigned taxonomic classifications. Likely contaminants and batch effects are removed. Feature selection methods can be used to select the most relevant features, which are used to train an ML model. Feature selection approaches are discussed in section “Methods for dimensionality reduction”. The model should be evaluated using an independent test set and cross-study validations, as discussed in section “Model validation”.

One of the basic aspects when characterizing the microbiome is its taxonomic composition. For that, it is necessary to assign DNA/RNA sequences to taxonomic units. A taxonomic unit is a set of related organisms, whose degree of relatedness depends on the type of unit chosen. It is useful to think of taxonomic units at the species level: in this case, relative abundance features are the proportion of organisms of each species in a sample^40^. A pipeline for cancer characterization using ML models based on abundance profiles, from sample collection to model validation, is shown in Fig. 2. Although some studies have analyzed microbiome profiles at the species level^41^, the broader genus level^18,42,43^, operational taxonomic units (OTUs), or the amplicon sequence variants (ASVs) are more frequently used^33,44^. OTUs group bacteria based on sequence similarity (usually at 97%), while ASVs identify true biological sequences (100% similarity) allowing the discrimination of closely related taxa. On one hand, it is less computationally demanding to determine OTUs or ASVs than to produce taxonomic classifications for each sequencing read^45^. On the other hand, the widely used target amplicon-based sequencing makes a species-level characterization of the microbiome difficult, leading to a preference for profiles at the genus level^46^. Alternatively, some authors have suggested using the abundances of multiple taxonomic levels as input features to an ML model^47,48^, or incorporating abundance profiles into phylogenetic trees^49^.

The microbial abundance data most often used when studying the links between the human microbiome and cancer has a set of characteristics that make the development of ML models challenging. Firstly, it has high dimensionality, as every taxon found in at least one sample constitutes a feature. Machine Learning models using a large number of features tend to overfit the training data, showing poor generalization to unseen samples^50^. As such, methods for dimensionality reduction are often advantageous when pre-processing microbiome data, prior to training ML models^51^. As a consequence of including one feature per taxon found, microbiome abundance profiles are sparse, with the abundances of a taxonomical unit typically following zero-inflated negative binomial distributions. This may impact the performance of ML and statistical models, leading to overdispersion^52^. The microbial abundance profiles of different samples are also usually obtained with variable sequencing depths. Thus, these data are compositional, as samples should not be characterized by the absolute abundance of each taxon, but rather by their relative counts^53^. However, it is unclear how this compositional framework impacts the performance of ML models^51^.

Current technologies used to characterize the microbiome allow the quantification of other variables besides the microbial composition profiles. These types of data can improve the performance of ML models over abundance profiles alone. As an example, in ref. ^54^, ML models trained with functional profiles to distinguish fecal samples from colorectal cancer and adenoma patients achieved greater accuracy than classifiers using taxonomical profiles. Similar results have been reported when predicting cachexia in lung cancer patients^55^ or the survival time of patients with neuroblastoma^31^. These functional profiles result from the quantification of the relative abundance of groups of organisms, genes, proteins, and metabolites^46,54^. Other types of microbiome data used as input for ML models in cancer-related applications include microbial single nucleotide polymorphisms^56^.

## Pre-processing microbial data

The data described in the previous section should be pre-processed to aid ML models in learning robust decision rules. Pre-processing steps may include transformations, which decrease the influence of outliers, normalization^57^, and feature selection; these will be discussed in this section. These pre-processing steps may greatly improve the performance of ML models. However, many ML models proposed for cancer characterization using microbiome data do not use any pre-processing other than data standardization^18,47,58^, as it is difficult to predict if such pre-processing steps will improve the performance of ML models before testing them.

### Methods for feature transformation

As previously mentioned, taxonomic profiles are heterogeneous, with an abundance of zero-valued features^59^. Thus, these are usually transformed to decrease the importance of dominant features and outliers, improving the performance of classifiers^60^. Log transformations (for which the transformed abundances ${x}^{{\prime} }$ are obtained from the original values *x* according to ${x}^{{\prime} }=log\left(x+c\right)$, with *c* known as the pseudocount, taking the value of one for most cases) are commonly used^61^; however, the chosen pseudocount value can impact the downstream analysis of the data, and it is unclear how to best choose this parameter^62^. Other transformations include a cube-root normalization proposed in Mulenga et al.^63^ and rescaling feature vectors to a unitary scale^48^. Mulenga et al.^63^ further proposed applying two normalization approaches to taxonomic profiles and concatenating the resulting features with the original values. This approach improved classification performance when combined with data augmentation with a variational autoencoder and a deep neural network^63^.

HARMONIES^59^ is a more complex transformation method developed for microbiome taxonomic data. This method uses Bayesian models to transform the read counts per taxon, taking different sequencing depths (the number of reads produced in each position in the sample genome) and data sparsity into account. It also considers that one feature may have zero abundance both because that taxon is absent from the sample, or because sequencing failed to capture it. However, HARMONIES^59^ has not yet been applied in conjunction with ML approaches. Despite the claims that these methods can better reflect the true microbiome or improve the accuracy of models, the most suitable normalization method is dependent on the original dataset: some transformations, such as those based on feature variance, are better at decreasing the influence of outliers, while normalization approaches preserving feature variance, as the cube-root, may be more effective when outliers are absent^60,63^.

### Methods for dimensionality reduction

Microbiome datasets have a large number of features, usually greater than the number of samples. Machine Learning models trained on data with a high number of features relative to the number of samples tend to overfit and show high variance, due to the large number of possible relationships exploited to create decision boundaries, most of them spurious^50^. Furthermore, training ML models in these settings is computationally expensive^64^. In these cases, it is recommended to use simpler models that are less likely to overfit the training data, with heavy regularization^50^; alternatively, methods for dimensionality reduction can be used. These include feature extraction methods, that transform the original data into a dataset with fewer features, and feature selection approaches, that select a subset of the original features for training^64,65^.

Most approaches to studying the link between cancer and the human microbiome using ML focus on the interpretability of models. Models are used to find biomarkers, such as species linked to a certain cancer type. Because of this, feature extraction methods such as principal component analysis (PCA) or principal coordinates analysis (PCoA), which transform the original feature space, are seldom used. Rather, most of the applied dimensionality reduction methods are filters for feature selection. These are independent of the model and act as a pre-processing step, selecting a subset of features based on the data alone. Typically, filter methods rank features according to a given criterion and select the highest-ranked^66^. These methods only consider the predictive power of one feature at a time, and not the discriminative capability of a set of features, as is the case for most ML models. As such, the features selected by filter methods may not form the optimal feature subset for the performance of the ML model.

Simple filter feature selectors use statistical tests to rank features according to their relevance. One such test is the Student’s *t* test for independent samples, which tests if a set of samples from two groups originated from distributions with the same mean. In feature selection for binary classification, samples are divided by class. The sample means for each feature and class are compared using a *t* test and the features with the highest statistic, reflecting a larger difference in means, are kept^50^. However, the *t* test should only be used when the features are approximately normally distributed. When this is not the case, it is possible to use a Mann-Whitney U test, assessing if the values of a feature are larger in one class than another. The Mann-Whitney U test is non-parametric and thus potentially more robust to outliers than the *t* test. Both tests were used in ref. ^44^ to find epithelial ovarian cancer from peritoneal fluid microbiota. For multiclass classification, one can resort to a one-way analysis of variance (ANOVA) test. One-way ANOVA tests compare the means of a feature between all classes using the F-distribution. In ref. ^54^, ANOVA was used to select features for colorectal cancer prediction from stool samples.

One downside of selectors based on statistical tests is their tendency to select many correlated features: if multiple features are correlated and have a large discriminative power, they are likely to all be selected despite being redundant^67^. Minimum Redundancy - Maximum Relevance (mRMR)^68^ was developed for microarray gene expression data. It selects uncorrelated and informative features. Reflecting its popularity, mRMR has been widely used in cancer characterization from microbiome data^69–71^. In mRMR, the feature relevance is estimated using information gain (IG) or an F-test between a feature and the target classes, for categorical/discrete and continuous variables, respectively. The first selected feature is that with the highest relevance criterium. For each subsequent iteration, the redundancy of the remaining unselected features is calculated through the sum of Pearson’s correlation coefficients or IG between the tested feature and all those previously selected. The algorithm then selects the features with the maximum ratio or difference of relevance to redundancy^68^.

Linear Discriminant Analysis Effect Size (LEfSe) is another popular method developed for feature selection on metagenomic data^72^. Besides statistical significance, LEfSe also takes biological relevance into account. It starts by discarding all features with *p* values greater than a threshold according to a Kruskall–Wallis test, which extends the Mann-Whitney U test to a multiclass setting. Thus, it selects the most relevant features for further analysis. Then, the dataset is further divided into sub-classes. These should reflect a biological characteristic of a sample (e.g. the sex of a patient, as done in ref. ^43^, or other clinical covariates in ref. ^33^). For each previously selected feature, all sub-classes in different classes are pairwise compared using a Wilcoxon signed-rank test. If any comparison results in a *p* value greater than a threshold, or if there is a difference in sign among the comparisons, the feature is deemed as not biologically robust and excluded. Lastly, Linear Discriminant Analysis is used to estimate the effect size of the resulting features and rank them^72^. LDA is trained by preserving classes as targets and using the original features along with covariates as input variables. The effect size is quantified by averaging the difference in class means with the difference in class means along the first linear discriminant axis^72^.

Autoencoders, which are discussed in greater detail in section “Autoencoders”, can also be used as filter feature selectors, despite having rarely been used in cancer-related host trait prediction from microbiome data.

Although used less frequently than filters due to the higher computational cost, feature selection can also be performed using embedded or wrapper methods and surrogate models. Tree-based models, such as Random Forests and Gradient-Boosted Trees can inexpensively give an importance score (such as Gini importance) to the input features^73^. The best-ranked features can be used as input data for a more powerful downstream model, responsible for the final classification^70^. However, the features selected by these surrogate models may not be the most adequate for the predictor, as these operate under different constraints and optimization approaches. Wrapper and embedded feature selection methods are dependent on the predictor model^74^. These include sparsity-aware approaches that set some of the model coefficients to zero, such as Least Absolute Shrinkage and Selection (LASSO) regression^75^, as used in refs. ^41,61,76^ and^47^, or Recursive Feature Elimination Support Vector Machines (SVM-RFE)^77^, which has been applied in ref. ^78^. Both approaches will be discussed in greater detail in the next section.

The most effective feature selection approach is dependent on the characteristics of the dataset, namely its sparsity and feature correlation, and the model used. One study compared the efficacy of statistical test-based methods, mRMR, a Fast Correlation Based Filter^79^, Conditional Mutual Information, and prior selection with Gradient Boosted Trees for dimensionality reduction in colorectal cancer prediction from metagenomic samples^70^. Statistical test-based methods, along with Gradient Boosted Trees selection achieved the best results when coupled with a Random Forest classifier; however, this may not hold true for other models or data: a feature selection approach should be selected on a case-by-case basis or with cross-validation. The authors also applied an ensemble selection, resulting in an 11% increase of AUC when compared to no feature selection^70^. Therefore, despite being computationally more expensive, ensemble approaches may provide more robust features selected by multiple different methods, and therefore suitable for a wider range of models.

Table 1 contains an overview of the methods discussed in this section, grouped by type (filter, wrapper, or embedded) along with the major advantages and disadvantages of each. In conclusion, despite being frequently used, it is unclear what feature selection method, if any, is best for each learning task. As such, feature selection approaches should be selected according to cross-validation. Alternatively, ensembles of selectors, or ML models capable of performing feature selection, such as deep learning approaches, may be used.

**Table 1:** Methods for microbiome feature selection

Type	Method	Advantages	Disadvantages	Examples
Filters	Statistical test based	Fast computation Selects relevant features	Selects redundant features Assumes feature independence Model agnostic	^54^
	mRMR	Avoids redundancy Selects relevant features	Assumes feature independence Model agnostic	^69–71^
	Surrogate model	Selects relevant features	Features may not be the best for the predictor Limited choice of surrogate models	^70^
	LEfSe	Takes biological relevance into account	Selects redundant features Assumes feature independence Model agnostic Requires biological covariates	^43^
Wrappers	RFE	Model-specific Selects relevant features	High computational cost Limited to some ML models	^78^
Embedded	LASSO	Fast computation Model-specific	Limited to some ML models	^41,44,47,61^

## Machine Learning models

Machine Learning models take a feature vector ${{{\bf{x}}}}\in {{\mathbb{R}}}^{M}$ as input, where *M* is the number of features, and predict a target value $\hat{y}$. This is done by applying a decision function *ϕ* such that $\hat{y}=\phi \left({{{\bf{x}}}}\right)$. Most ML models try to minimize a loss function that quantifies the difference between the predicted and ground-truth variables^80^. Learning tasks can be divided into classification or regression, when the target variable is categorical or numerical, respectively^75^. When studying the cancer-related microbiome, classification is the most common, mainly encompassing cancer diagnosis or tumor type identification. However, regression tasks can also be found in literature, as in survival time prediction^31^ or predicting the number of tumors formed in murine models^81^.

In this section, we will discuss the most popular and promising ML methods applied to cancer identification and characterization from the microbiome. Table 2 shows some examples of these applications and lists the ML models used in each.

**Table 2:** Examples of applications of ML methods for cancer characterization from microbiome data

Learning task	SVM	Decision trees	Random forest	Boosted trees	Logistic regression	NNs
Cancer type identification	^69^	–	^69^	^18^	–	–
Identification of colorectal cancer	^48,56,71,85,89,91^	^85,91,154^	^28,48,56,76,85,89,91,154^	^56,85,91^	^76,85,91^	^48,56,57,61,61,63^
Identification of other cancer types	^87,94,155^	^87^	^33,44,58,87,98,108,156–159^	^43,160,161^	^43,44,87,155,160,162,163^	^87^
Multi-disease identification (including cancer)	^41,164–166^	–	^41,165–168^	^169^	^41,164^	^47,165,166,169^
Prediction of treatment outcomes and survival time	^32,32,90^	^32,150^	^31,32,90,149^	–	^32,151^	^32,149^
Identification of cancer precursors and complications	^92^	^92^	^35,42,55,92^	–	^92^	^92^

### Support Vector Machines

Support Vector Machines (SVMs)^82^ are commonly used with microarray data^83^. Therefore, these are also often employed to identify microbiome-related biomarkers for cancer. In their simplest form, SVMs are binary classification models that define the decision boundary as a hyperplane. When the data is linearly separable, there are infinite hyperplanes that perfectly separate the samples according to their class^75^. SVMs select the hyperplane with the largest margin to the training data^84^ and only some of the training samples define the decision boundary; these are called the support vectors^80^.

Linear SVMs were found to achieve comparable performance to non-linear models in colorectal cancer identification from microbial abundance profiles^85^. Nevertheless, SVMs can be adapted to non-linear decision boundaries by extending the original feature space with feature transformations^50^, which results in non-linear decision boundaries in the original feature space and improves separability. Feature extension can be done without increasing the computational load with the so-called kernel trick^86^. The most used kernel in tumor-associated microbiome analysis is the radial basis function (RBF)^85,87^, which has achieved good results in colorectal cancer prediction^48,71^. However, when kernels are used, the prediction for a sample is based on its kernel distance to the support vectors^88^. Therefore, the decisions of SVMs are more difficult to interpret than with Random Forests or Logistic Regression^88^.

SVMs have been used to predict colorectal patient survival time^89,90^, cancer prognosis, and drug responses^78^ from microbiome and gene expression data, and to identify colorectal cancer patients using taxonomic profiles^71,85^. Furthermore, SVMs are frequently used as benchmarks when evaluating other methods^47,87,91^. The popularity of SVMs for cancer-related host trait prediction from the microbiome is due to their widespread availability in ML libraries and the good performance demonstrated by these models in multiple works. In^41^, SVMs outperformed Logistic Regression when predicting various diseases using taxonomic profiles. In a few cases, SVMs achieved better or comparable results to Random Forrest classifiers^41,48^. However, in most of the experimental settings, SVMs failed to surpass the performance of Random Forest, namely in survival time prediction^90^, identification of cancer types^69^, and colorectal polyp identification^92^.

Because SVMs are binary classifiers, extending them to multiclass classification comes with multiple problems. Usually, a classifier is trained for each class, in a one-versus-rest approach, with the final prediction taken as the one yielding the highest value for the decision function. Because each classifier is trained with different target labels, there is no assurance that these values are scaled equally^80^. Furthermore, this approach is computationally expensive, as it requires training a potentially large number of classifiers. These issues make SVMs rarely used for microbiome-based multicancer prediction. In such an application, an SVM achieved a much worse performance than a Random Forest, a k-Nearest Neighbors, and a Decision Tree classifier^69^, all models that can be directly used in multiclass settings.

Despite all the aforementioned shortcomings, SVMs may prove useful when only a small training set is available. These models are less prone to overfit the training data and can show good generalization performance when the number of features far exceeds that of training samples^93^. This has motivated its usage in oral carcinoma identification from taxonomic and functional profiles in a study comprising only 38 samples^94^. However, it is unclear if other models could have achieved comparable performance, as no other approaches were tested. Furthermore, this good generalization performance is dependent on an adequate choice of the regularization parameter controlling the width of the decision boundary margins, which requires costly cross-validation^75^.

Using a wrapper, SVMs can be adapted to perform feature selection. As previously mentioned, this is known as Recursive Feature Elimination Support Vector Machine (SVM-RFE)^77^. In SVM-RFE, an SVM is initially trained on all the features. In each iteration, the feature with the smallest contribution according to the model parameters is removed, and the SVM is retrained^77^.

### Decision Tree-based models

Models based on Decision Trees - Random Forests in particular - are the most frequently used ML models when studying the link between cancer and the human microbiome. Decision Trees are sequential models that apply successive rules to yield a final prediction. At each level of the tree, starting from the root, the value of a feature is compared to a threshold learned from the training data. The decision path followed by the model is defined by the result of each comparison and the final prediction is given by the class associated with the leaf reached^95^. When splitting a node, Decision Trees exhaustively search for the threshold and feature achieving the best purity in the child nodes^50^. Purity, expressing the homogeneity in target classes of the samples in a leaf, is commonly quantified using the Gini index or entropy^96^.

#### Random Forests

Decision Trees are prone to exhibit high variability, as small changes in the training data may result in different splitting thresholds. Alterations in the first nodes propagate into changes in the structure of the subsequent levels, greatly impacting the overall classification rules^50^. This leads to high variance and helps explain why Decision Trees are rarely used when studying the link between cancer and the microbiome. Bagging decreases the variance shown by Decision Trees: multiple models are trained using samples and features randomly sampled with replacement from the training set^50,97^. Bagging Decision Trees results in a Random Forest; the different trees have slightly different structures, as they were trained on different data^95^. The final prediction is the average of the predictions of the individual trees^95^.

In a work on multicancer identification from microbial abundances, a Random Forest classifier achieved improved generalization performance over a Decision Tree^69^. A similar result was found in ref. ^85^ for colorectal cancer identification. Unlike Decision Trees, Random Forest classifiers are highly popular when studying the association between cancer and the microbiome. This is supported by several published benchmarking experiments in which this model outperformed all other tested algorithms in tasks including identifying colorectal cancer^85^, melanomas in mice^87^, cancer subtypes^69^, and other host traits^41^. Random Forests have been applied to predicting the survival time of colorectal cancer patients from gene expression and microbiome taxonomic profiles^90^ and identifying several tumor types such as epithelial ovarian cancer^44^, tonsillar squamous cell carcinoma^58^, lung adenocarcinoma^33^, colorectal cancer^28^, oral squamous cell carcinoma^98^, and in a multiclass classification setting^69^.

#### Boosting

Boosting is another approach to decrease variance and operates on a similar idea to bagging; however, instead of training the standalone estimators independently, it builds models sequentially^80^ and these are kept shallow^99^. When boosting Decision Trees, each tree is trained with samples drawn from the dataset, weighted according to the performance of the previous classifier on that data point. This way, the next individual model is trained to improve the performance on data that the previous tree failed to predict^80^. The final prediction is taken as a weighted average of the prediction of all the trees, or as a majority voting^80^. The improved generalization performance of boosting methods over Decision Trees was also exemplified in ref. ^85^. However, it is unclear whether boosting can achieve better performance over Random Forests in cancer-related host trait prediction from the microbiome. In this same publication, a boosting approach and a Random Forest demonstrated comparable performance^85^, while in ref. ^87^ a boosting model showed a decreased AUC but improved precision and recall over Random Forests. Boosting methods have also been proposed to predict tissue malignancy in breast cancer using bacterial taxonomic profiles from biopsies^30^ and to identify several tumor subtypes from microbiome data^18^.

#### Interpretability and Explainable Boosting Machines

Besides the excellent performance demonstrated by Random Forests and boosting models in cancer-related microbiome host trait prediction, these models are often used due to their improved interpretability over other *black box* algorithms such as non-linear SVMs and Neural Networks. For sufficiently shallow Decision Trees, decision rules can be easily analyzed and understood, making finding putative biomarkers easier. Decision tree flowcharts are already widely used in medicine for diagnosis, so they are familiar to doctors and health professionals^100^. As Random Forests and boosting combine the predictions of several trees, this direct interpretation of feature contribution is lost. However, it is possible to quantify the importance of each feature by analyzing the number of times a feature is used in a node or the average increase in purity achieved with it^73^. In ref. ^42^, Random Forests were used to identify acute myeloid leukemia from taxonomic profiles, and the contribution of each taxon for classification was assessed using the average increase in purity. However, these scores do not fully reflect the complex decision rules of the classifier, which depend on the association of multiple features.

With boosting, the link between importance scores and decision rules is even more unclear than for Random Forests, as different trees have a different impact on the final prediction. Explainable Boosting Machines (EBM)^101^ try to make this association clearer so that the decision of a model is better understood through feature importance scores. EBM is an implementation of Generalized Additive Models^102^ that also models pairwise feature interactions, and in which the feature transformations are modeled using a boosting algorithm. When training EBMs, each tree can only use one feature. The set of *M* features is iterated multiple times, with each tree contributing by a small amount to the final classification. Because of this, a large number of trees is needed in EBMs, making training times longer than for other boosting approaches^103,104^. The importance of each feature can be easily assessed by analyzing the importance scores and using heatmaps for pairwise interactions^104^. Because each tree can use only one feature and is a weak classifier, these scores provide better insight into the classification rules^101^. Explainable Boosting Machines have been used to identify colorectal cancer and adenoma from microbial gut functional profiles^54^, allowing greater interpretability without sacrificing accuracy. Glass-box methods such as EBM may help identify novel biomarkers for cancer, based on the human microbiome.

### Logistic Regression

Logistic Regression is a linear model for classification that, due to its simplicity, has been widely used in studies investigating the tumor-specific microbiome, mainly for feature selection and benchmarking^47,61^. This model assumes that the microbiome data originated from class-conditional Gaussian distributions of equal covariance matrices. The decision function of Logistic Regression, shown in Eq. (1) for binary classification, is the estimated posterior probability of a sample **x** being of a given class^75^. The model parameters *β* and *β*_0_ are learned using iterative processes, usually gradient descent with binary cross entropy as a loss function^75^.

1 $$\phi \left({{{\bf{x}}}}\right)=\frac{1}{1+{e}^{{{{{\bf{x}}}}}^{T}\beta +{\beta }_{0}}}$$

Despite having linear decision boundaries, Logistic Regression classifiers can, in some cases, achieve similar results to non-linear counterparts in microbiome cancer-related host trait prediction: in ref. ^85^, L2 regularized Logistic Regression achieved a comparable AUC to Random Forest and Boosted Trees for colorectal cancer identification. However, this is seldom observed^49,87,91^; as examples, Logistic Regression failed to improve colorectal polyp identification over a Multilayer Perceptron and Naïve Bayes classifier^92^, and colorectal cancer identification over a Multimodal Neural Network^47^. Because of this decreased performance, Logistic Regression has mainly been used for feature selection.

L1 and L2 regularization are approaches to decrease model variance by constraining its parameters^105^. L2 regularization penalizes the squared value of the model parameters (**β** and *β*_0_), decreasing their absolute values^106^. L1 regularization, also known as Least Absolute Shrinkage and Selection Operator (LASSO), constrains the model parameters so that the sum of their absolute values is less than a number^105^. LASSO regression forces some of the parameters to take null values, effectively disregarding the corresponding features during inference^50^. Combining L1 and L2 regularization results in a model known as ElasticNet^107^. Therefore, LASSO and ElasticNet are embedded forms of feature selection and have been used to select microbiome features for a more powerful downstream classifier. As the decision boundary is linear, feature contribution for classification can more intuitively be quantified by the corresponding model parameter. The most relevant features are chosen accordingly^44,61^. This approach was used for colorectal cancer identification coupled with a Multilayer Perceptron^57^ and Generalized Neural Networks^61^. LASSO was also included in the ensemble used for feature selection in ref. ^44^ for ovarian cancer identification from taxonomic profiles and serum tumor marker levels.

The same properties that make Logistic Regression adequate for feature selection also explain its improved interpretability over methods such as Random Forests and Artificial Neural Networks: the contribution of each feature can be quantified by the parameters of the model^85^. This allows researchers to more easily identify potential bacterial biomarkers associated with specific types of cancer^85^. This strategy was used to identify seven genera potentially associated with invasive cervical cancer^108^ and to access feature contributions when identifying colorectal cancer using taxonomic profiles^85^.

### Artificial Neural Networks

Artificial Neural Networks (NNs) are complex non-linear ML models that usually achieve better performance than their simpler counterparts^109^. To build an NN, multiple activation units are stacked into layers. Activation units take a vector as input and output a scalar, applying an activation function on the linear combination of inputs and a bias parameter. The outputs of a layer are concatenated and fed into the following layer until the last is reached. This last layer is the output layer, as it outputs the final prediction of the model. The layers between the input and output are called hidden layers^110^. Architectures with more than one hidden layer are considered Deep Neural Networks^110^. The model parameters are learned using stochastic gradient descent. Because the gradients for the parameters of each activation unit are computed using the chain rule from the output to the input layer, this computation is referred to as back-propagation^110^.

Unlike other ML methods, Deep NNs do not require extensive previous feature selection and transformation. These models are complex enough to do the necessary processing of input features^111^. The output vectors of hidden layers can be perceived as mappings of a sample to a transformed feature space, in which class separability is enhanced^110^. This transformation can be exploited for dimensionality reduction and data augmentation, as we will discuss briefly. Nevertheless, multiple works on the tumor-specific microbiome have used Deep NNs trained with previously selected features^48,87,92^. Doing so may hinder performance, as these features may not be ideal given the model.

Because of their complexity and non-linearity, the decision rules of NNs are difficult to understand. Thus, NNs are seen as *black box* models that make biomarker identification more difficult than Random Forests or linear models. In host trait prediction from microbiome data, works assessing feature importance usually resort to model-agnostic methods: Mulenga et al.^63^ used L2 regularization, while Arabameri et al.^61^ employed Derivative-based Feature Selection. These methods do not provide a complete understanding of the model decisions, nor of the interdependencies between features, as provided by Decision Trees, for instance.

#### Multilayer perceptrons

**Fig. 3:**
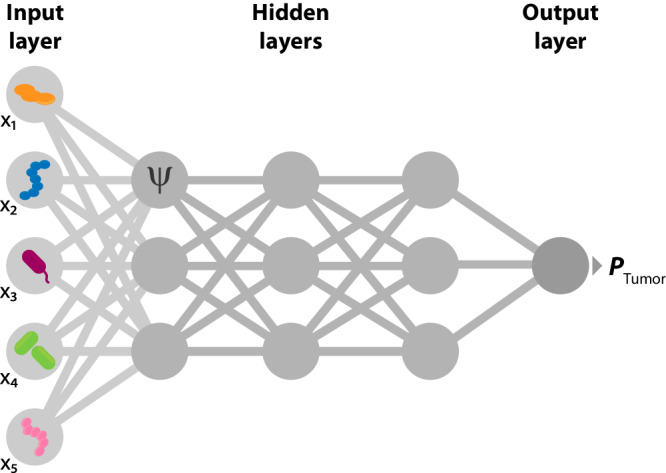
Diagram of an MLP for cancer prediction from microbial abundance profiles. The input layer contains the relative abundance of each taxon or OTU. These act as inputs (**x**) for the activation units in the hidden layers, which apply a function *ψ*. The output layer returns the probability of the presence and absence of cancer.

The Multilayer Perceptron (MLP) is an NN in which consecutive layers are fully connected, as seen in Fig. 3. Multilayer Perceptrons can easily overfit the training data if no regularization is adopted, such as dropout and batch normalization, due to their large number of parameters^112^. In ref. ^48^, an MLP failed to improve the performance of an RBF-kernel SVM for colorectal cancer prediction using microbiome taxonomic profiles. However, the MLP used features pre-selected using Linear Regression, which may not be ideal for NN classification. Similar findings have also been reported for melanoma identification in mice while employing feature selection^87^. On the other hand, the MLP with prior feature selection used in ref. ^61^ achieved higher accuracy but worse sensitivity when compared to a *k*-Nearest Neighbors classifier and an SVM. Therefore, it is unclear if MLPs can achieve better predictions than those provided by simpler models such as Random Forests and non-linear SVMs.

One notable approach using MLPs is that of DeepEn-Phy, a model operating on relative abundances incorporated into a phylogenetic tree^49^. This model uses a set of cascaded MLPs trained end-to-end. The phylogenetic trees are divided into levels, from leaves to roots. Each MLP operates in one level, with a level having multiple MLPs. The first MLPs use relative abundances in the leaf nodes and their outputs are fed into downstream MLPs, until reaching the root MLP. This model achieved better results when predicting smoking status and body mass index using the gut microbiome than Random Forest, Gradient Boosted Trees, and PopPhy-CNN, another deep learning model operating on phylogenetic trees^113^.

#### General Regression Neural Networks

General Regression Neural Networks (GRNN)^114^ are variations of kernel regression networks^115^. In a GRNN, each of the *N* activation units applies an RBF centered in each of the training samples during inference. The output of the model is a weighted sum of the activation units^114^. One advantage of GRNN over MLPs is the smaller number of hyperparameters: only the spread parameter needs to be specified, while in MLPs one must choose the number of layers and activation units, and the optimization parameters^61^. However, the pattern layer grows with the training set, making learning and inference computationally expensive^116^.

This model was used to detect colorectal cancer from taxonomic profiles of the gut microbiome, having outperformed an MLP and an RBF kernel SVM^61^. According to the authors, GRNNs can achieve better accuracy than MLPs, even with a small number of training samples.

#### Multimodal Neural Networks

Multimodal NNs combine MLPs to integrate features from different modalities. In one cancer and microbiome-related application, a multimodal NN was used to identify several diseases, including colorectal cancer, using taxonomic and functional profiles from the gut microbiome^47^. MLPs were trained using features from each modality alone. Embeddings were derived by concatenating the outputs from the last hidden layers of each modality. The multimodal NN achieved improved accuracy over MLPs that used just one of the feature types; it also outperformed Random Forest, an SVM, Gradient Boosted Trees, and LASSO regression for colorectal cancer prediction^47^. This work shows the potential of combining multiple types of data in increasing the accuracy of tumor diagnosis and introduces multimodal NNs as a promising method to integrate various modalities of features that complementarily capture the complex mechanisms that underly cancer development.

#### Autoencoders

The outputs of hidden layers of NNs can be interpreted as mappings of the input vectors into a latent space. If a layer has less than *M* activation units, with *M* the number of input features, these can be used as compressed representations of samples^75^. Autoencoders are NNs that try to reconstruct the input data. Their goal is not to achieve useful predictions; rather, autoencoders learn representations of the input vectors in a latent space^110^.

Due to the high dimensionality of microbiome data, autoencoders can be useful in avoiding the curse of dimensionality. However, despite having been proposed for dimensionality reduction in metagenomic studies^117^, autoencoders are rarely used in cancer-related host trait prediction from the microbiome. In ref. ^118^, autoencoders coupled with an MLP failed to improve the AUC in identifying colorectal cancer from the gut microbiome over MetAML, which uses Random Forests, SVMs, LASSO regression, or an ElasticNet^118^. However, it is unclear if this is due to the classifier used or the dimensionality reduction strategy.

Autoencoders can be adapted to generate synthetic samples. Because data augmentation techniques increase the number of training samples, they decrease the variance of a model, assuming that the synthetic samples are derived from a similar distribution to the original data^119^. This is particularly important for models that are prone to overfit the training data, such as deep NNs. While studying the cancer-related microbiome, this issue is made worse due to the scarcity of data. Therefore, data augmentation can be a powerful aid in the development of more accurate ML models acting on microbiome data. In ref. ^63^, data augmentation using Variational Autoencoders (VAE) improved the predictions of an NN classifier for colorectal cancer using gut microbial abundances^63^, with an increase in the AUC of up to 30%.

### Choosing the right ML model for the task

The best choice for a microbiome-based ML model for cancer-related applications depends on the diversity, quality, and quantity of the available data and on the learning task. Table 3 lists the main strengths and weaknesses of ML models discussed in this review and is intended to aid researchers in selecting an ML model for their particular use cases. Nevertheless, it is difficult to know a priori which model will achieve the best performance, and testing multiple models is recommended.

**Table 3:** Advantages and disadvantages of ML methods for cancer characterization from microbiome data

Type	Method	Advantages	Disadvantages
SVM	SVM	Can be adapted for wrapper feature selection Good generalization performance Good performance with few samples Widely available implementations	Difficult interpretation of rules High sensitivity to hyperparameter tuning Not adequate for multiclass classification Computationally expensive
Tree-based	Decision Tree	Easy to interpret Widely available implementations	High variance
	Random Forest	Improved interpretability over SVMs and NNs Low variance Widely used Usually outperforms other methods Widely available implementations	Reduced interpretability over Decision Trees and Logistic Regression
	Boosted Trees	Improved interpretability over SVMs and NNs Low variance	Computationally expensive Reduced interpretability over Random Forests and Logistic Regression
Logistic Regression		Can be adapted for embedded feature selection (LASSO) Easy to interpret Widely available implementations	Linear decision boundary
Artificial Neural Networks		High complexity Large variety of architectures Prior feature selection is not necessary Can generate new data Promising results for improved performance over other methods Aid data integration	Very difficult to interpret Computationally expensive to train Few works using them so far Require large numbers of samples

As previously mentioned, Random Forests are highly popular, as they achieve a good balance between interpretability and performance for most applications. When inference performance is of the utmost importance (for instance, in cancer screening), more complex methods such as Boosted Trees or NNs may be needed. However, this implies a higher computational cost for training when compared to other, more simple models. The decision rules of Boosted Trees and NNs are also difficult to interpret, making it difficult to validate the decisions of the models and to identify characteristics of the microbiome associated with a given prediction. Furthermore, in the case of deep NNs, any increase in performance and generalizability over Random Forests and other approaches requires large amounts of training data, which are usually not available in cancer-associated microbiome studies. When the datasets available for training are small, SVMs are likely a good choice^93,94^; however, these models require costly hyperparameter tuning and do not support multiclass classification, hindering their application for multicancer identification^80^.

When the goal is to identify microbiome-based cancer biomarkers, being able to derive meaning from the decisions of the ML models is more important than achieving the best performance possible. Therefore, in these use cases, simpler and more interpretable models (e.g. Decision Trees and Logistic Regressors) may be the best choice. Both Decision Trees and Logistic Regressors have widely available implementations and their decision rules are easy to analyze. However, Decision Trees tend to show high variance and poor generalizability^50^, while Logistic Regressors are limited to linear decision boundaries, which may be unsuited for some learning tasks^92^.

## Model validation

After training an ML model, its performance should be properly evaluated to ensure its generalizability and to avoid biases that are not related to the microbiome nor clinically relevant, such as those caused by technical artifacts. Models should be tested with a hold-out dataset, independent of that used for training. Including information about the training data in the test dataset, also known as data leakage, prevents an exact assessment of how the model will behave with previously unseen data. This can lead to an overestimation of model performance and generalizability^120^. Thus, when studying the links between cancer and the human microbiome, ML model validation should ideally include evaluating the model performance using data generated in a slightly different manner (e.g., from another institution) than that used for training, and excluding patients in the training set. When such data is not available, cross-validation can be used as an alternative. In cross-validation, the training dataset is split into *k* subsets of the same size. The ML model is trained and evaluated *k* times, each time using a different subset for validation and the remaining *k* − 1 for training. The performance metrics are then averaged to yield a final estimate of the performance of the model.

The same care should be taken when selecting hyperparameters. To ensure that the selected values are those that maximize the performance of a model with previously unseen data, these should be selected through cross-validation^121^.

It is key to also avoid data leakage in the feature transformation and dimensionality reduction steps, as any inference about the structure of the data based on the test set or the entire dataset may compromise generalizability. As an example, if using LEfSe with microbial abundance profiles, only the abundances of samples in the training dataset should be used to evaluate statistical significance and effect sizes. However, promising bacterial biomarkers are often chosen through statistical tests on the whole dataset, before being used as input features to an ML model. This feature selection based on all samples may lead to the overfitting of the dataset and poor generalizability. In other cases, the publications are not clear about the validation strategies employed and the possibility of data leakage, making it difficult to assess the generalization of their conclusions.

## Current limitations and future perspectives

**Fig. 4:**
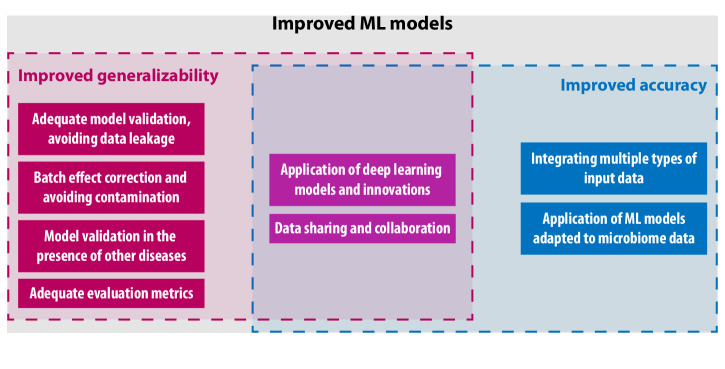
Overview of needed improvements in microbiome-based identification of cancer. Improvements in ML models can be achieved through improvements in their accuracy and generalizability. Some of the future perspectives discussed in this review and shown in this figure can aid in improving generalizability (left), model accuracy (right), or both (center).

In this section, we discuss the current limitations of research on ML methods for microbiome-based cancer characterization, along with how to address them. Future perspectives in this area are also provided here. Figure 4 contains an overview of the limitations discussed in this section.

Many publications on ML for cancer characterization from microbiome data have contradictory results, with questionable generalization. As an example, in refs. ^71^ and ^28^, the genus *Fusobacterium* was found to be more abundant in the gut microbiome of colorectal cancer patients; however, this genus was not identified as a possible biomarker in refs. ^18,27^ nor ^61^. Zhou et al.^29^ aggregated the proposed microbial biomarkers for colorectal cancer from various publications and found inconsistent results. Nevertheless, the overall findings show a difference between colorectal cancer and the healthy microbiome, although the microorganisms responsible may differ across studies. The findings in ref. ^18^ have also been questioned with models said to be relying on bacteria unlikely to be present in humans to predict cancer types^122,123^; however the authors have since reproduced their findings with a revised methodology^124,125^. These conflicting results are caused by three major factors: an inadequate validation of ML models; the usage of small datasets to train and evaluate ML models; and a lack of correction of technical variations.

Besides the lack of agreement in microbial biomarkers, these shortcomings make it difficult to compare the impact of different ML algorithms and pre-processing approaches in model performance - an improvement may not be a result of a different methodology, but due to overfitting. Recently, some studies have focused on improving the generalizability of ML models to detect colorectal cancer, with multiple datasets from different regions and a consistent processing pipeline^18,27^. However, this should be extended to all works using ML.

### Inadequate ML model validation and the need for larger datasets

In section “Model validation”, we have discussed how to properly evaluate the performance of an ML model, with a focus on ensuring its generalizability. However, as previously mentioned, not all research adheres to these recommendations. Most notably, data leakage is frequent during the feature transformation and dimensionality reduction steps, leading to an overestimation of model performance with unseen data and potentially to non-generalizable results.

Furthermore, to achieve sufficient model generalizability, researchers should ensure that the dataset used during development reflects the characteristics of the population the model will be used on. For instance, any model trained with fecal microbiome data of patients of a specific geographic region is likely to perform poorly for patients of a different area, as geography is known to impact the composition of the gut microbiome^126^. Using large datasets from diverse populations can therefore lead to more generalizable models and in turn to approaches that can be used with a greater number of patients.

However, the creation of these large datasets is costly and time-consuming. Therefore, it is crucial to implement data-sharing protocols between researchers, ensuring that data from patients across the world are integrated into the development and validation of ML models. When possible, collected microbiome data should be made publicly available in online repositories such as NCBI’s SRA^127^. Alternatively, processed data can also be hosted online, although this limits the ways in which these data can be repurposed. Furthermore, researchers should be transparent about how the microbiome data used in their studies was generated and processed, and how models were developed and validated. All code used should be made publicly available, facilitating future analyses and allowing for a more thorough assessment of model validation. Together, these approaches can improve the generalizability of ML models and accelerate the discovery of further links between the human microbiome and cancer.

Generative ML models can also aid in expanding the available datasets through the generation of synthetic data. These models are often based on NNs and include the Autoencoders discussed in section “Autoencoders” and Generative Adversarial Networks (GANs)^128^. However, and despite recent breakthroughs in generative ML models for genomics^129^, these have seldom been used to uncover the association between cancer and the human microbiome.

### The effects of technical variations and clinical covariates

The lack of correction for batch effects and data leakage further raises doubts about how the findings of most works can be generalized. Not all differences in the data are linked to biologically relevant processes: rather, some may be due to technical variation or clinical covariates. As an example, the TCGA dataset is known to show strong batch effects linked to sequencing centers^18,130–132^. In these cases, ML models may be relying on these non-biologically relevant patterns for their predictions. This leads to poor generalizability and puts the findings derived from these models into question.

When it is possible to normalize the data to remove technical variations, this should be done. However, in some cases, the biological variable of interest is perfectly correlated with another covariate. Under these conditions, it is almost impossible to determine which changes are biologically relevant. Therefore, this ambiguity should be avoided, or, at least, control samples enabling the normalization of the data should be acquired. Testing models with hold-out datasets, obtained under different conditions than the ones used to train the models can also avoid spurious associations between cancer and traits of the microbiome by flagging ML models with poor generalizability.

Knowing when an ML model is relying on technical artifacts to make accurate predictions is often difficult due to the *black box* nature of most models. For this reason, more interpretable models, such as Decision Tree-based approaches, should be preferred. Recent advances in *post hoc* explainability^133–135^ may also enable the assessment of more complex decision rules, such as those from deep NNs. With these advances, researchers can analyze how ML models make decisions, detecting when these are relying on patterns that are unlikely to be related to biological phenomena.

### Unclear predictive capability of ML models based on microbiome data

The predictive capability of ML models applied to the tumor-specific microbiome is still unclear. For colorectal cancer identification from gut taxonomic profiles, classifiers can currently achieve a promising performance: the GRNN in ref. ^61^ had an AUC of up to 85% in an independent test set, while the SVM in ref. ^71^ achieved 81% accuracy while using microbial abundance features. These metrics are indicative of good performance, however, they are unlikely to be robust enough for widespread use yet. Care should also be taken regarding the evaluation metrics used, to provide representative estimates of model performance. Analyzing the performance of models is difficult due to the small datasets used for development^18,43^, especially for cancer types other than colorectal, which in some cases have as few as 23 patients^30^. Furthermore, models are often evaluated using a one-versus-rest approach, which exacerbates class imbalance. Evaluation metrics such as accuracy are not adequate for highly imbalanced datasets. Even the AUC may hide the low precision or recall of a classifier, with a heavily skewed class distribution^136^. For instance, despite having an AUC of over 82%, in many cases reaching 100%, the classifiers to distinguish between cancer subtypes using the tumor microbiome in ref. ^18^ have an Area Under the Precision-Recall Curve (AUPR) of as little as 36%. To better understand the potential of ML approaches in identifying tumors based on the microbiome, these should be evaluated using metrics adequate for imbalanced datasets such as AUPR and the F-score^136^.

Improving the predictive power of ML models applied to cancer microbiome analysis will likely require the implementation of state-of-the-art methods such as NNs, increased regularization, and the aforementioned development of larger bespoke datasets. Despite the increasing popularity of NNs for this purpose, many advances in deep ML architectures are still to be applied. For instance, Autoencoders or attention mechanisms^137^ are seldom used, despite having achieved improved performance over other methods in biological tasks^138,139^. We expect these approaches to better model the complex relations between cancer and the human microbiome. However, when using these methods, special care should be taken to avoid overfitting. As such, the current preference for simpler ML models may be explained by a lack of data, which further highlights the need for larger datasets. Furthermore, new ways to constrain the latent representations of the input vectors are needed to avoid overfitting.

### Unclear specificity of microbial biomarkers with confounding diseases

It is still unclear if bacterial biomarkers for cancers found using ML models hold specificity in the presence of other diseases. Most of the published research on the tumor-specific microbiome compares the microbiome profiles of cancer patients to those of healthy individuals, excluding those with other diseases^71,98^. Works developing models capable of identifying other diseases, such as type 2 diabetes or IBD, only include colorectal cancer^41,47^. Excluding confounding diseases that may affect the microbiome is likely to make it simpler to find alterations linked to cancer. However, these will not be adequate for widespread clinical usage in case they lose their predictive power in the presence of comorbidities. Type 2 diabetes increases the risk of cancer^140,141^, so employing biomarkers that cannot distinguish between the two diseases may yield incorrect results for a large fraction of the human population. Several publications have also found that type 2 diabetes is linked to disruptions in the gut microbiome^142^. Therefore, it is unlikely that the patterns differentiating the tumor and healthy microbiomes will be similar to those found between cancer and type 2 diabetes. Further research into the differences between the human microbiota in the presence of tumors and other confounding diseases is required to better understand the limitations of using microbiome-derived biomarkers for cancer diagnosis and analysis.

### Microbiome features other than taxonomic profiles are largely unused

The vast majority of ML approaches take on taxonomic abundance profiles as input data. However, these may not be the most informative characteristics of the microbiome: for instance, gut microbiome functional profiles were shown to provide an improvement in performance over the taxonomic counterparts when distinguishing colorectal cancer from adenoma^54^. Data integration (for instance, using both taxonomic and functional features, as done in^47^, or coupling the taxonomic profiles with other methods for cancer diagnosis such as fecal occult-blood testing^71^) may also improve the classification accuracy in cancer diagnosis, but few publications exploit this for tumors other than colorectal.

The emergence of next-generation sequencing (NGS), allowing high-throughput sequencing and shotgun metagenomics, has enabled the cost-effective functional profiling of the microbiome through technologies such as mRNA sequencing (RNA-Seq)^143,144^. Epigenomics analyses were also made possible by NGS^144^. Therefore, we expect further work to be undertaken to better understand how these different types of data and biological characteristics relate to one another, and if data integration can yield more powerful methods for cancer diagnosis and characterization.

### The need for ML models adapted to the characteristics of microbiome data

The relationships between the host and its microbiome are highly complex, and microbiome data has characteristics that make ML models challenging. For once, abundance taxonomic profiles have many zero values and small variations linked to technical phenomena. This leads to the most common ML approaches overfitting the training data. Thus, the next steps in ML for cancer-related applications using microbiome data likely include the development and application of models that are more robust under zero-inflated data with high variability. Several models have already been proposed for this kind of data^145–147^, some specifically designed for microbial count data^148^. Such models should find microbial signatures associated with cancer characteristics while filtering out sporadic associations resulting from technical variations. In conjunction with this, we expect further work on feature selection and dimensionality reduction approaches for microbial count data to improve the performance of ML models for cancer characterization from microbiome data. Again, by preserving only the most biologically relevant features, these approaches could reduce the variability of models.

### Translation to clinical practice and ethical concerns

Although the performance of ML methods for cancer characterization using microbiome data is currently insufficient for clinical use, it is important to delineate how future models can be incorporated into clinical workflows. So far, most works have focused on discovering microbial biomarkers through the use of ML. These biomarkers, after careful validation, could potentially be the target of diagnostic assays. Blood or stool-derived microbial signatures, in particular, are promising, as these would enable a non-invasive screening of high-risk patients^18^. Patients exhibiting these biomarkers could then be further examined through imaging, histologic, and cytologic exams to arrive at a diagnosis. Microbiome-based biomarkers could also be used to guide treatment by predicting the efficacy of different approaches^32,35,149–151^. All decisions and diagnoses derived from microbial biomarkers should be discussed and carefully explained to patients, using a patient-centered approach. To this end, it is crucial that patients are aware of the shortcomings of these biomarkers and the uncertainties surrounding the links between cancer and the microbiome.

Although these non-invasive methods could allow for better care and improved survival rates, clinicians are often wary of diagnostic tools derived from ML models due to their *black box* nature^152^. This highlights the need for clinicians to be involved in the development of these tools and for ML models with more understandable decision rules. Furthermore, we should avoid increasing the workload of clinicians when integrating these approaches into the clinical workflow^152^.

Finally, the ethical use of ML in cancer diagnosis and treatment requires stringent measures to safeguard patient privacy and ensure data security. These should include anonymization, encryption, and transparency to guarantee that the identities and health information of patients remain protected^153^. This is also important for the regulatory approval of tools integrating ML algorithms using microbiome data. Therefore, balancing patient confidentiality and safety with innovation will require adapting existing frameworks to accommodate the dynamic nature of these technologies and their evolving clinical implications.

### The need for interdisciplinary approaches

As we have seen, ensuring adequate ML model development and translating these models or their findings to clinical practice is a complex and interdisciplinary endeavor: proper model training and testing requires familiarity with computer science, as expert knowledge is needed to avoid data leakage and to extract understandable insights into the decision rules of ML models. An inadequate understanding of these concepts may lead to overly optimistic estimates of model accuracy and the generalizability of findings. Likewise, biologists should be involved in model validation (especially in the assessment of their decision rules), ensuring that these make reasonable predictions and are not relying on clinically irrelevant artifacts. Furthermore, clinical and microbial field knowledge may also aid in the development of the ML model itself. Finally, clinicians should be a part of model development and validation, ensuring its applicability. This interdisciplinary approach should be fostered and the norm rather than the exception.

## Conclusions

The human microbiome holds great potential for cancer diagnosis, prognosis, and therapies, offering a promising avenue for research. However, the biomarkers derived from this complex system present a challenge in their analysis. To address this, ML methods have emerged as crucial tools and have been widely used in recent breakthroughs to unravel cancer-microbiome relationships. In this review, we described and compared current ML approaches from sample collection to the final prediction. Most approaches have focused on gut-derived taxonomic profiles, mainly for colorectal cancer prediction. Tree-based models are the most used and recommended when there is a need to understand the decision rules of models; however, recent works suggest that deep learning architectures such as VAEs and multimodal NNs can achieve better performance and offer a great tool for applications requiring excellent inference performance, such as diagnosis. Many published works do not ensure the generalization of their models, avoiding data leakage and confounding technical variables, leading to conflicting results. Therefore, future research should be careful to implement adequate generalization assessments with expanded hold-out datasets and careful removal of technical variations.

Despite promising, models developed for cancer types other than colorectal do not yet achieve sufficient performance for clinical usage. Improving this performance will likely require integrating microbial taxonomy profiles with other data types, including gene expression and host characteristics, leveraging advances in sequencing technology. This approach will be critical for a better understanding of the complex interplay between the microbiome and the human host in the context of cancer. We also expect the recent advances in deep learning approaches, along with the application of ML methods better suitable to the characteristics of microbiome data to aid in this endeavor. Finally, data-sharing and interdisciplinary efforts are instrumental in ensuring the development of accurate and generalizable models.

### Reporting summary

Further information on research design is available in the Nature Research Reporting Summary linked to this article.

### Supplementary information

Supplementary Material

Reporting summary

## Data Availability

The full-text articles assessed in this review are listed in Supplementary Table 1.
