# Supplementary material for: A review of machine learning methods for cancer characterization from microbiome data
Source: NPJ Precis Oncol. 2024 May 30;8:123. doi: 10.1038/s41698-024-00617-7 (PMC11139966; doi:10.1038/s41698-024-00617-7)
Supplement: Supplementary file 1 — Supplementary Material [file 41698_2024_617_MOESM1_ESM.pdf]

Supplementary Methods 1 – Criteria for Inclusion

We searched for relevant research articles in Scopus, PubMed, and IEEEXplore.

Scopus was queried with the entry:

```
TITLE-ABS-KEY(machine AND learning AND (tumor OR neoplasia) AND (microbiome OR bacteria OR microbiota)) AND (LIMIT-TO(PUBSTAGE, "final")) AND (LIMIT-TO(DOCTYPE, "ar") OR LIMIT-TO(DOCTYPE, "cp")).
```

PubMed was queried with the entry:

```
((((microbiota) OR (microbiome)) OR (bacteria)) AND (machine learning)) AND (((tumor) OR (neoplasia)) OR (cancer))
```

IEEEXplore was queried with the entry:

```
("All Metadata": microbiome OR "All Metadata": bacteria OR "All Metadata": microbiota) AND ("All Metadata": tumor OR "All Metadata": cancer OR "All Metadata": neoplasia) AND ("All Metadata": machine learning)
```

In all cases, we filtered for articles published between 01/01/2015 and 01/05/2023.

Abstracts deemed relevant mentioned the application of a Machine Learning method for cancer diagnosis or characterization (e.g., stage or survival time). Estimation of other clinical variables related to cancer, such as treatment outcomes, was also considered relevant. Works not using ML methods but proposing or testing pre-processing approaches suitable for ML models, or novel ML architectures designed for metagenomic analysis were also included. Meta-analysis of studies using ML for biomarker identification in cancer were also included. Abstracts for which it was unclear if an ML model was used were kept and assessed during full-text review.

Criteria for full-text inclusion follows closely those for abstracts. Reviews and perspective articles were excluded. Relevant works included the application of a Machine Learning method for cancer diagnosis or characterization (e.g., stage or survival time), either as a main focus or as a further experiment on previously identified biomarkers or proposed metagenomic techniques. Estimation of other clinical variables related to cancer, such as treatment outcomes, was also considered relevant. Works not using ML methods but proposing or testing pre-processing approaches suitable for ML models, or novel ML architectures designed for microbiome analysis were also included. Meta-analysis of studies using ML for biomarker identification in cancer were also included.

We analyzed full-text articles focusing on the application of ML methods for biomarker discovery and cancer diagnosis and characterization. Articles were classified according to:

1. The ML model used.
2. The learning task (the variable to predict).
3. The type of sample from which microbiome data was derived.
4. The type of input features and data used to train the ML model.
5. The feature selection method used, if any.

Furthermore, we kept note of the most relevant findings relating to the application of ML methods to the tumor-specific microbiome. Supplementary Table 1 contains the results of this classification.

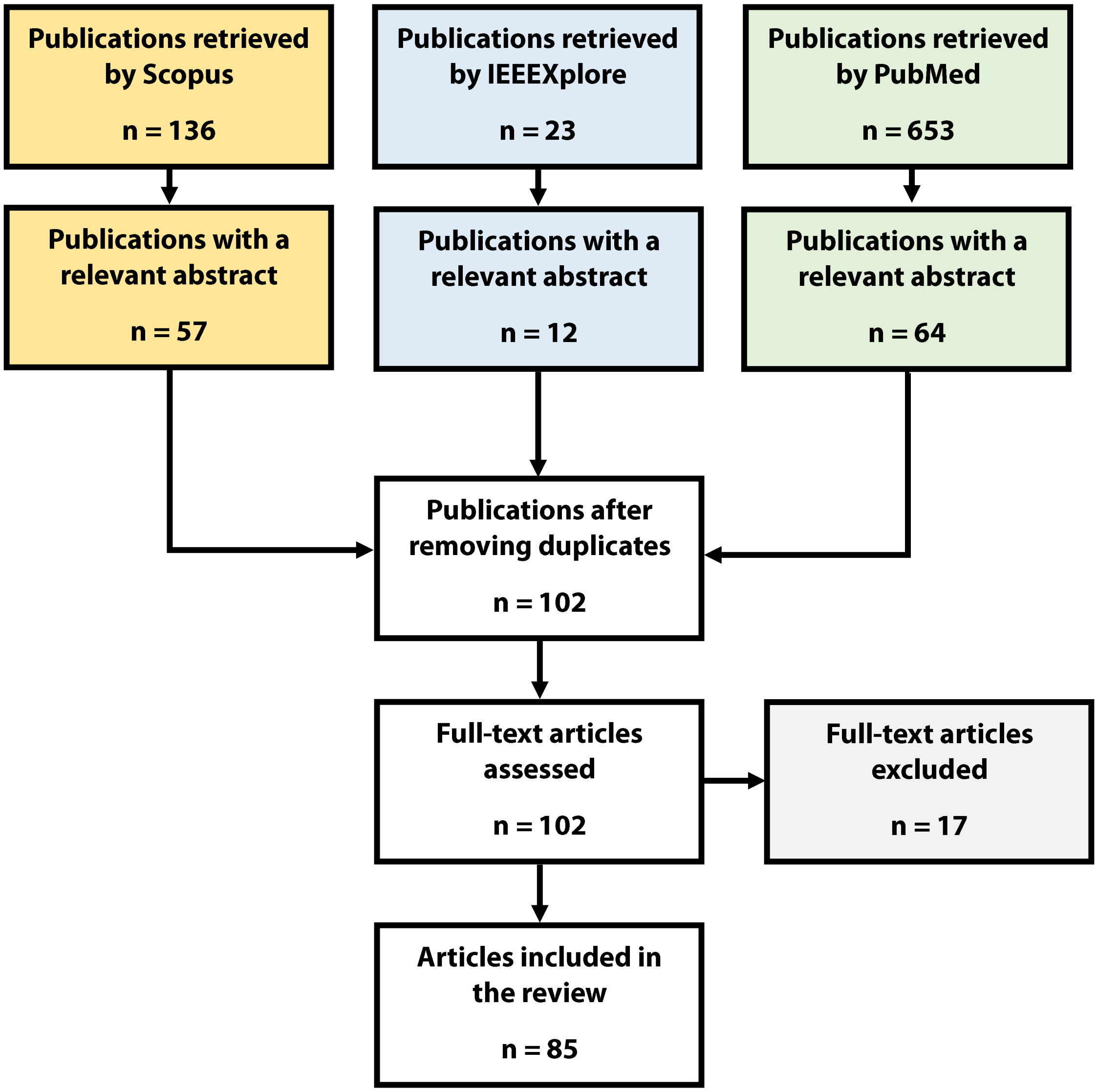

Supplementary Figure 1 – Chart for full-text article selection and review.

Supplementary Table 1 - Full-text articles included

Title	Author	ML Model	Learning Task	Input Features	Sample Type	Feature Selection Method	Notes	Search Method
A Hybrid Machine Learning Approach for the Phenotypic Classification of Metagenomic Colon Cancer Reads Based on Kmer Frequency and Biomarker Profiling	Kishk et al.	Random Forest and RBF-kernel SVM	Identifying colorectal cancer	Taxonomical Profiles and k-mer Frequencies	Biopsy	None	Using k-mer frequencies results in a decrease of performance when compared with taxonomical profiles.	IEEE Xplore
Deep ensemble learning over the microbial phylogenetic tree (DeepEn-Phy)	Ling et al.	DeepEn-Phy (MLPs) and PopPhy-CNN* (Convolutional NN)	Predicting smoking status and Body Mass Index	Taxonomical Profiles as Phylogenetic Trees	Fecal	None		IEEE Xplore
Enhancing Metagenome-based Disease Prediction by Unsupervised Binning Approaches	Nguyen et al.	MLP	Identifying colorectal cancer, liver cirrosis, obesity, HIV, type 2 diabetes, and IBD	Taxonomical Profiles	Fecal	None	Comparison of normalization and feature discretization approaches.	IEEE Xplore
Evaluation of the oral microbiome as a biomarker for early detection of human oral carcinomas	Warnke-Sommer & Ali	SVM	Identifying oral carcinoma	Taxonomical and Functional Profiles	Oral swabs	None		IEEE Xplore
Feature Extension of Gut Microbiome Data for Deep Neural Network-Based Colorectal Cancer Classification	Mulenga et al.	MLP	Identifying colorectal cancer	Taxonomical Profiles	Fecal	VAE and Feature Extension	Comparison of normalization approaches. Proposes cube root normalization for abundance data.	IEEE Xplore
Host trait prediction from human microbiome data for Colorectal Cancer	Ashraf et al.	Logistic Regression, kernel SVM, Naïve Bayes, Decision Tree, Random Forest, Bagged Decision Trees, AdaBoost, Ensamble, Extra Tree, k-fold Random Forest, Gradient Boosted Trees	Identifying colorectal cancer	Taxonomical Profiles	Biopsy	None	Ensamble methods generaly outperform stand-alone classifiers.	IEEE Xplore
Human Gut Microbiome Data Analysis for Disease Likelihood Prediction Using Autoencoders	Wickramaratne et al.	Random Forest, SVM, MLP	Identifying colorectal cancer, liver cirrosis, obesity, type 2 diabetes, and IBD	Taxonomical Profiles	Fecal	Autoencoder		IEEE Xplore
Identifying Taxonomic Biomarkers of Colorectal Cancer in Human Intestinal Microbiota Using Multiple Feature Selection Methods	Jabeer et al.	Random Forest	Identifying colorectal cancer	Taxonomical Profiles	Fecal	mRMR, Fleuret's CMIM, FCBF, XGBoost, Information Gain and other statistical tests	Comparison of feature selection methods. Demonstrates that feature selection aids classification.	IEEE Xplore
Stacking and Chaining of Normalization Methods in Deep Learning-Based Classification of Colorectal Cancer Using Gut Microbiome Data	Mulenga et al.	MLP	Identifying colorectal cancer	Taxonomical Profiles	Fecal	LASSO	Comparison of normalization approaches.	IEEE Xplore
Archaea Microbiome Dysregulated Genes and Pathways as Molecular Targets for Lung Adenocarcinoma and Squamous Cell Carcinoma	Uzelac et al.	Gradient Boosted Trees	Identifying lung adenocarcinoma and lung squamous cell carcinoma	Taxonomical Profiles	Biopsy	RFE	Models were interpreted using SHAP values	PubMed
Bacterial Alterations in Post-Cholecystectomy Patients Are Associated With Colorectal Cancer	Ren et al.	Random Forest	Identifying post-cholecystectomy patients	Taxonomical Profiles	Fecal	None		PubMed
Carcinogenesis of Male Oral Submucous Fibrosis Alters Salivary Microbiomes	Chen et al.	Random Forest	Identifying oral squamous cell carcinoma in patients with oral submucous fibrosis	Taxonomical Profiles and clinical covariates	Saliva	LEfSe		PubMed
Colonic Microbial Abundances Predict Adenoma Formers	Watson et al.	Random Forest	Identifying adenoma formers	Taxonomical Profiles	Fecal, oral, non-adenoma, and adnoma-adjacent mucosa	None		PubMed
Different Characteristics in Gut Microbiome between Advanced Adenoma Patients and Colorectal Cancer Patients by Metagenomic Analysis	Han et al.	Random Forest, Gradient Boosted Trees, SVM, MLP, CatBoost	Distinguishing colorectal cancer from advanced adenoma	Viral abundances and bacterial SNPs	Fecal	RFE	Bacterial SNPs outperformed viral abundances in regards to accuracy	PubMed
DMFMDA: Prediction of Microbe-Disease Associations Based on Deep Matrix Factorization Using Bayesian Personalized Ranking	Liu et al.	MLP with input embedding	Identifying the association between bacteria and colon cancer	Taxa and disease types	None	None	Deep learning approach for matrix factorization	PubMed
Establishment and evaluation of prediction model for multiple disease classification based on gut microbial data	Bang et al.	k-Nearest Neighbors, Logistic Model Trees, Additive Logistic Regression, and SVM	Identifying multiple sclerosis, juvenile idiopathic arthritis, myalgic encephalomyelitis/chronic fatigue syndrome, acquired immune deficiency syndrome, stroke, and colorectal cancer	Taxonomical Profiles	Fecal	RFE	Trained multiclass classifiers	PubMed
Exploring Gut Microbiome in Predicting the Efficacy of Immunotherapy in Non-Small Cell Lung Cancer	Liu et al.	Random Forest and MLP	Predicting the efficacy of immunotherapy in Non-Small Cell Lung Cancer patients	Taxonomical, funcitonal, and protein profiles	Fecal	None	Objective framed as a binary classification task, for progression-free survival greater than 6 months or less than 3 months	PubMed
Faecal microbiome-based machine learning for multi-class disease diagnosis	Su et al.	Random Forest, K-Nearest Neighbours, MLP, SVM, and Graph Convolutional NN	Identifying colorectal cancer, colorectal adenomas, Crohn's disease, ulcerative colitis, irritable bowel syndrome, obesity, cardiovascular disease, and post-acute COVID-19 syndrome	Taxonomical Profiles	Fecal	None	The Random Forest model outperformed all others in terms of AUC	PubMed
Host DNA contents in fecal metagenomics as a biomarker for intestinal diseases and effective treatment	Jiang et al.	Random Forest	Identifying colorectal cancer and Crohn's disease, and redicting the response to terapy of Crohn's disease patients	Taxonomical and Functional Profiles and host DNA content	Fecal	None		PubMed
iCEMIGE: Integration of CEll-morphometrics, MIcrobiome, and GEne biomarker signatures for risk stratification in breast cancers	Mao et al.	Multivariate Cox Regression and CMS-ML	Predicting overall survival and progress-free survival rates	Taxonomical Profiles, Gene Expression, and Histological Images	Biopsy	Multivariate Cox Regression		PubMed
Identifying distinctive tissue and fecal microbial signatures and the tumor-promoting effects of deoxycholic acid on breast cancer	Wang et al.	Random Forest	Identifying breast cancer	Taxonomical Profiles	Fecal and breast tissue	RFE		PubMed
Improve the Colorectal Cancer Diagnosis Using Gut Microbiome Data	Zhou et al.	Random Forest and Bayesian Additive Regression Trees	Identifying colorectal cancer	Taxonomic Profiles	Fecal	Hierarchical feature engineering and AUC-RF		PubMed
Interpretable and accurate prediction models for metagenomics data	Prifti et al.	Binary/Ternary/Ratio, Linear and RBF-SVM*, Random Forest*, and Logistic Regression*	Identifying multiple diseases	Taxonomical and Functional Profiles, and Marker Genes	Variable	Statistical tests	Propose Binary/Ternary/Ratio for classification and regression, which is a simpler version of a linear model. Used 54 public datasets	PubMed
Distinct composition and metabolic functions of human gut microbiota are associated with cachexia in lung cancer patients	Ni et al.	Random Forest	Identifying cachexia in lung cancer patients	Taxonomical and Metabolic Profiles	Plasma	Statistical Tests		Scopus
Manipulation of the Gut Microbiota Reveals Role in Colon Tumorigenesis	Zackular et al.	Random Forest Regressor	Predicting the number of tumors formed on a murine model after antibiotic treatment	Taxonomical Profiles	Fecal	None	Regression task. Used OTU abundances before modulation of the microbiome with antibiotics to predict the number of tumors developed in mice	PubMed

Meta-analysis of fecal viromes demonstrates high diagnostic potential of the gut viral signatures for colorectal cancer and adenoma risk assessment	Chen et al.	Random Forest and LASSO	Identifying colorectal cancer and adenoma	Viral taxonomical profiles	Fecal	None		PubMed
Metagenomic Analysis of Common Intestinal Diseases Reveals Relationships among Microbial Signatures and Powers Multidisease Diagnostic Models	Jiang et al.	Random Forest	Identifying IBD, colorectal cancer, and ulcerative colitis	Taxonomical and Functional Profiles	Fecal	None and statistical tests		PubMed
Predicting cancer immunotherapy response from gut microbiomes using machine learning models	Liang et al.	Logistic Regression, LASSO, ElasticNet, Decision Tree, Random Forest, MLP, SVM and an ensamble of all	Predicting the efficacy of immunotherapy in cancer patients	Taxonomical Profiles	Fecal	None	Learning task framed as a binary classification problem	PubMed
Pretreatment gut microbiome predicts chemotherapy-related bloodstream infection	Montassier et al.	Random Forest	Predicting the occurence of bacteremia in linfoma patients after chemotherapy	Taxonomical Profiles	Fecal	None		PubMed
Signatures of Mucosal Microbiome in Oral Squamous Cell Carcinoma Identified Using a Random Forest Model	Zhou et al.	Random Forest	Identifying oral squamous cell carcinoma	Taxonomical Profiles	Biopsy	RFE		PubMed
Specific gut microbiome signature predicts the early-stage lung cancer	Zheng et al.	SVM and Logistic Regression	Identifying lung cancer	Taxonomical Profiles	Fecal	Statistical Tests, mRMR, and RFE		PubMed
Taxonomic and Functional Dysregulation in Salivary Microbiomes During Oral Carcinogenesis	Chen et al.	Random Forest	Identifying oral cancer	Taxonomical and Functional Profiles	Saliva	RFE		PubMed
The Intratumoral Bacterial Metataxonomic Signature of Hepatocellular Carcinoma	Huang et al.	Random Forest	Identifying hepatocellular carcinoma	Taxonomical Profiles	Liver and peritumor tissue	Random Forest		PubMed
The salivary metatranscriptome as an accurate diagnostic indicator of oral cancer	Banavar et al.	Logistic Regression	Identifying oral cancer	Taxonomical and Functional Profiles	Saliva	Variance Filter and Bootstrap Logistic Regression		PubMed
Vaginal Microbiome-Based Bacterial Signatures for Predicting the Severity of Cervical Intraepithelial Neoplasia	Lee et al.	Random Forest	Identifying cervical intraepithelial neoplasia	Taxonomical Profiles	Vaginal smear	RFE		PubMed
A Framework for Effective Application of Machine Learning to Microbiome-Based Classification Problems	Topçuoglu et al.	Logistic Regression, L1, L2, and RBF-kernel SVM, Random Forest, Gradient Boosted Trees, Decision Tree	Identifying colorectal cancer	Taxonomical Profiles	Fecal	None	Compared classifiers, implementing good ML practices and analyzing interpretability.	Scopus
A multi-omics machine learning framework in predicting the survival of colorectal cancer patients	Yang et al.	Random Forest, kernel SVM, and Naïve Bayes	Predicting the survival time of colorectal cancer patients	Taxonomical Profiles and Transcriptomics (differential gene expression)	Biopsy	None	Framed the problem as a binary classification task, in which > 3 years was considered long-term survival.	Scopus
A reliable method for colorectal cancer prediction based on feature selection and support vector machine	Zhao et al	MLP, kernel SVM, Naïve Bayes, Random Forest, and k-Nearest Neighbors	Identifying colorectal cancer	Taxonomical Profiles and clinical covariates	Fecal	Logistic Regression assuming independent features	The highest accuracy was obtained using an RBF-kernel SVM.	Scopus
Assessment of peritoneal microbial features and tumor marker levels as potential diagnostic tools for ovarian cancer	Miao et al.	LASSO regression, Random Forest	Identifying ovarian cancer	Taxonomical Profiles and Serum Tumor Marker Levels	Peritoneal Fluid	Ensamble of Random Forest, LASSO regression, t-test, Distance Corelation, and Mann-Whitney U Test		Scopus
Biopsy bacterial signature can predict patient tissue malignancy	Hogan et al.	Gradient Boosted Trees	Identifying breast cancer	Taxonomical Profiles	Biopsy	None		Scopus
Machine-learning algorithms for asthma, COPD, and lung cancer risk assessment using circulating microbial extracellular vesicle data and their application to assess dietary effects	McDowell et al.	Gradient Boosting Machine (GBM), Generalized Linear Model, MLP and a GBM + MLP ensamble	Identifying chronic obstructive pulmonary disease, asthma, and lung cancer	Accumulated taxonomical profiles from extracellular vesicles	Serum	Statistical Tests		PubMed
Circulating microbial content in myeloid malignancy patients is associated with disease subtypes and patient outcomes	Woerner et al.	Random Forest	Identifying acute myeloid leukemia and destinguishing its percursors	Taxonomical Profiles	Blood and Bone Marrow	None		Scopus
Detection of Colorectal Carcinoma Based on Microbiota Analysis Using Generalized Regression Neural Networks and Nonlinear Feature Selection	Arabameri et al.	General Regression Neural Network, SVM*, Random Forest*, k-Nearest Neighbors*, Naïve Bayes*, and MLP*	Identifying colorectal cancer	Taxonomical Profiles and Fecal Occult Blood Test results	Fecal	Derivative-based Feature Selection and LASSO Regression		Scopus
Development and evaluation of a colorectal cancer screening method using machine learning-based gut microbiota analysis	Konishi et al.	Stacked Ensamble	Identifying colorectal cancer	Taxonomical Profiles, age, and body mass index	Fecal	None		Scopus
Prediction of breast cancer using blood microbiome and identification of foods for breast cancer prevention	An et al.	Logistic Regression and Gradient Boosting Machine	Identifying breast cancer	Taxonomical Profiles	Serum	LEfSe, statistical tests, and RFE		Scopus
Distinct microbial communities colonize tonsillar squamous cell carcinoma	De Martin et al.	Random Forest	Identifying tonsilar cell carcinoma	Taxonomical Profiles	Biopsy	None		Scopus
Distinct tumor bacterial microbiome in lung adenocarcinomas manifested as radiological subsolid nodules	Ma et al.	Random Forest	Identifying lung adenocarcinomas and distinguishing between solid and subsolid nodules	Taxonomical Profiles	Biopsy	LEfSe		Scopus
Dynamics of Fecal Microbiota with and without Invasive Cervical Cancer and Its Application in Early Diagnosis	Kang et al.	LASSO regression, Random Forest	Identifying invasive cervical cancer	Taxonomical Profiles	Fecal	Recursive Feature Elimination		Scopus
Fecal Bacteria as Non-Invasive Biomarkers for Colorectal Adenocarcinoma	Yuan et al.	Kernel SVM	Identifying colorectal cancer	Taxonomical Profiles	Fecal	mRMR		Scopus
Gut Microbial Shifts Indicate Melanoma Presence and Bacterial Interactions in a Murine Model	Rossi et al.	MLP, RBF-kernel SVM, Random Forest, AdaBoost, CN2 Rule Inducer, k-Nearest Neighbors, Naïve Bayes, Decision Tree, Logistic Regression	Identifying melanoma in mice	Taxonomical Profiles	Fecal	PCA	The MLP and Random Forest exhibited the best F1-score and AUC, respectively.	Scopus
Gut microbiome identifies risk for colorectal polyps	Dadkhah et al.	Naïve Bayes, Decision Tree, Logistic Regression, Random Forest, k-Nearest Neighbour, MLP, SVM	Identifying colorectal polyps	Taxonomical Profiles	Biopsy, fecal, rectal swabs	Kruskal-Wallis, MetaStats, LEfSe, Indicator		Scopus
HARMONIES: A Hybrid Approach for Microbiome Networks Inference via Exploiting Sparsity	Jiang et al.	Bayesian Modeling	Normalizing taxonomical profiles	Taxonomical Profiles	Fecal	None	Proposed a novel method for microbial abundance data normalization	Scopus

Human Gut Microbiome-Based Knowledgebase as a Biomarker Screening Tool to Improve the Predicted Probability for Colorectal Cancer	Zhou et al.	None	Identifying colorectal cancer	Taxonomical Profiles	Fecal	None	Aggregated the proposed biomarkers of several studies into a database.	Scopus
Identifying Robust Microbiota Signatures and Interpretable Rules to Distinguish Cancer Subtypes	Chen et al.	Random Forest, SVM, k-Nearest Neighbors, and Decision Tree	Identifying cancer subtypes	Taxonomical Profiles	Biopsy	mRMR and Recursive Feature Selection		Scopus
Leveraging sequence-based faecal microbial community survey data to identify a composite biomarker for colorectal cancer	Shah et al	Random Forest	Identifying colorectal cancer	Taxonomical Profiles	Fecal	None		Scopus
Machine Learning Meta-analysis of Large Metagenomic Datasets: Tools and Biological Insights	Pasolli et al.	Random Forest, SVM, LASSO regression, and ElasticNet	Identifying colorectal cancer, liver cirrosis, obesity, type 2 diabetes, and IBD	Taxonomical Profiles	Fecal and Swabs	Random Forest	Proposed the MetAML tool for metagenomics-based diagnosis.	Scopus
Microbiome analyses of blood and tissues suggest cancer diagnostic approach	Poore et al.	Gradient Boosted Trees	Identifying cancer subtypes	Taxonomical Profiles	Biopsy and blood	None	Proposed a method for cancer identification based on blood microbial DNA. Created a dataset of microbial abundances from TCGA data.	Scopus
Multimodal deep learning applied to classify healthy and disease states of human microbiome	Jae Lee & Rho	Multimodal Neural Network, Random Forest*, XGBoost*, LASSO regression*, Principal Component Regression*, and SVM*	Identifying IBD, type 2 diabetes, liver cirrhosis, and colorectal cancer	Taxonomical and Functional Profiles	Fecal	None	Integrating functional and taxonomical data improved the classification performance when compared to single-mode MLPs.	Scopus
Predictable modulation of cancer treatment outcomes by the gut microbiota	Heshiki et al.	Decision Tree	Predicting cancer treatment outcomes	Taxonomical and Functional Profiles	Fecal	Statistical tests		Scopus
Predicting cancer immunotherapy response from gut microbiomes using machine learning models	Liang et al.	Decision Tree, Logistic Regression, MLP, SVM	Predicting cancer treatment outcomes	Taxonomical Features	Fecal	None		Scopus
Taxonomy dimension reduction for colorectal cancer prediction	Qu et al.	Random Forest, Decision Trees, and Naïve Bayes	Identifying colorectal cancer	Taxonomical Profiles	Fecal	Correlation-based Feature Selection and Maximum Relevance Maximum Distance	Compared feature selection methods. Correlation-based Feature Selection achieved the highest AUC.	Scopus
The Clinical Potential of Oral Microbiota as a Screening Tool for Oral Squamous Cell Carcinomas	Zhou et al.	Random Forest	Identifying oral squamous cell carcinoma	Taxonomical Profiles	Saliva, subgenvival plaque, tumor surface, and mucosa swabs, and biopsy	None		Scopus
The Machine-Learning-Mediated Interface of Microbiome and Genetic Risk Stratification in Neuroblastoma Reveals Molecular Pathways Related to Patient Survival	Li et al.	Random Forest	Predicting the survival time of neuroblastoma patients	Taxonomical Profiles	Biopsy	None		Scopus
Towards a metagenomics machine learning interpretable model for understanding the transition from adenoma to colorectal cancer	Casimiro-Soriguer et al.	Explainable Boosting Machine	Identifying colorectal cancer	Taxonomical and Functional Profiles	Fecal	ANOVA		Scopus
A Deep Learning Approach to Predict Health Status Using Microbiome Profiling	Wickramaratne et al.	MLP	Identifying colorectal cancer, obesity, and underweight patients	Taxonomical Profiles	Fecal	None	Used species and genus-level profiles	IEEE Xplore
A multi-task learning method for analyzing microbiota as cancer immunotherapy signal	Jiang et al.	Multi-task Neural Network, MLP*, SVM*, Random Forest*, Logistic Regression*, Naïve Bayes*	Predicting immunotherapy treatment outcomes in non-small cell lung cancer and renal cell carcinoma	Taxonomical Profiles	Fecal	None	The Multi-task Neural Network is made up of two MLPs sharing information in the third hidden layer	IEEE Xplore
Automatic disease prediction from human gut metagenomic data using boosting GraphSAGE	Syama et al.	Ensamble of Graph Neural Networks, SVM*, MLP*, Random Forest*, Deep Forest*, and Gradient Boosted Trees*	Identifying colorectal cancer and inflammatory bowel disease	Taxonomical Profiles	Fecal	None	The Graph Neural Network outperformed all other models. The input graph was built using the similarity between the taxonomical profiles of samples.	Scopus
Brain tumor diagnostic model and dietary effect based on extracellular vesicle microbiome data in Serum	Yang et al.	Logistic Regression and Gradient Boosting Machine	Identifying brain cancer	Taxonomical Profiles	Serum and Biopsy	Akaike Information Criterion, LEfSe, Gradient Boosting Machine		Scopus
Leveraging explainable AI for gut microbiome-based colorectal cancer classification	Rynazal et al.	Random Forest	Identifying colorectal cancer	Taxonomical Profiles	Fecal	Abundance thresholds	Used SHAP values to explain the decisions of the models	Scopus
Breast cancer patients from the Midwest region of the United States have reduced levels of short-chain fatty acid-producing gut bacteria	Shrode et al.	Random Forest	Identifying breast cancer	Taxonomical Profiles	Fecal	None		Scopus
Global Meta-analysis of Urine Microbiome: Colonization of Polycyclic Aromatic Hydrocarbon-degrading Bacteria Among Bladder Cancer Patients	Bukavina et al.	Logistic Regression	Identifying bladder cancer	Taxonomical Profiles	Urine	Abundance thresholds		Scopus
A non-antibiotic-disrupted gut microbiome is associated with clinical responses to CD19-CAR-T cell cancer immunotherapy	Stein-Thoeringer et al.	Logistic Regression	Predicting the response to CAR-T cell therapy	Taxonomical Profiles, age, and gender	Fecal	Lasso	Used baseline (prior to treatment) profiles as input features	Scopus
Untangling determinants of gut microbiota and tumor immunologic status through a multi-omics approach in colorectal cancer	Zahang et al.	Logistic Regression and SVM	Identifying colorectal cancer	Taxonomical Profiles	Fecal	RFE		Scopus
The Role of Intratumor Microbiomes in Cervical Cancer Metastasis	Jiang et al.	Random Forest, SVM, and Generalized Linear Models	Predicting cervical cancer metastasis	Taxonomical and Gene Expression Profiles	Biopsy	Statistical tests		Scopus
Prediction model of poorly differentiated colorectal cancer (CRC) based on gut bacteria	Qi et al.	Logistic Regression, Random Forest, MLP, Gradient Boosted Trees, and SVM	Distinguish poorly differentiated from moderately differentiated colorectal cancer	Taxonomical Profiles	Fecal	None		Scopus
Cross-cohort gut microbiome associations with immune checkpoint inhibitor response in advanced melanoma	Lee et al.	Logistic Regression	Predicting the response to immune checkpoint inhibitor treatment	Taxonomical and Functional Profiles	Fecal	None		Scopus
A Goldilocks Principle for the Gut Microbiome: Taxonomic Resolution Matters for Microbiome-Based Classification of Colorectal Cancer	Armour et al.	Random Forest, Logistic Regression, XGBoost, Gradient Boosted Trees, and SVM	Identifying colorectal adenomas and carcinomas	Taxonomical Profiles	Fecal	None	Compared the performance of ML models using different taxonomic levels. Family, genus, and OTU levels achieved the best results	Scopus
Alterations of the Human Lung and Gut Microbiomes in Non-Small Cell Lung Carcinomas and Distant Metastasis	Lu et al.	Random Forest	Identifying non-small cell lung carcinoma	Taxonomical Profiles	Fecal and sputum	Statistical tests and RFE		Scopus
Normalization of the microbiota in patients after treatment for colonic lesions	Sze et al.	Random Forest	Identifying pre and post-treatment patients with colorectal carcinoma and adenoma	Taxonomical Profiles	Fecal	None		Scopus
Systematic evaluation of supervised classifiers for fecal microbiota-based prediction of colorectal cancer	Ai et al.	All models available in the Weka 3.6.13 package for Java	Identifying colorectal cancer	Taxonomical Profiles	Fecal	None		Scopus

Intratumoral Microbiota-Host Interactions Shape the Variability of Lung Adenocarcinoma and Lung Squamous Cell Carcinoma in Recurrence and Metastasis	Zhou et al.	Random Forest, Naïve Bayes, Gradient Boosted Trees	Predicting recurrence and metastasis of lung cancer	Taxonomical Profiles and Host Gene Expression	Biopsy	LEfSe and Statistical Tests		PubMed
DeepGeni: deep generalized interpretable autoencoder elucidates gut microbiota for better cancer immunotherapy	Oh and Zhang	Random Forest, SVM, MLP	Predicting response to treatment of melanoma patients	Taxonomical Profiles	Fecal	Autoencoder	Applied data augmentation with DeepBioGen (based on Generative Adversarial Networks). The importance of the latent features was assessed with Extremely Randomized Trees and propagated through the model weights into the input space.	PubMed
Leveraging Scheme for Cross-Study Microbiome Machine Learning Prediction and Feature Evaluations	Song and Zhou	Logistic Regression, Random Forest, and SVM	Identifying colorectal cancer and Crohn's disease, and predicting immunotherapy response	Taxonomical Profiles	Fecal	Boruta		PubMed
Enhanced metagenomic deep learning for disease prediction and consistent signature recognition by restructured microbiome 2D representations	Shen et al.	AggMapNet (CNN)	Identifying cirrhosis, obesity, type 2 diabetes, inflammatory bowel disease, and colorectal cancer	Taxonomical Profiles	Fecal	MEGMA	Propose a method (MEGMA) to represent abundance data as 2D colored images	PubMed
Prediction model of colorectal cancer (CRC) lymph node metastasis based on intestinal bacteria	Yinhang et al.	Logistic Regression, Random Forest, MLP, Gradient Boosted Trees, and SVM	Identifying colorectal cancer lymph node metastasis	Taxonomical Profiles	Fecal	None		PubMed

* Model used for comparison and not the main focus of the study
